# CDK5 Regulates Paclitaxel Sensitivity in Ovarian Cancer Cells by Modulating AKT Activation, p21Cip1- and p27Kip1-Mediated G1 Cell Cycle Arrest and Apoptosis

**DOI:** 10.1371/journal.pone.0131833

**Published:** 2015-07-06

**Authors:** Shu Zhang, Zhen Lu, Weiqun Mao, Ahmed A. Ahmed, Hailing Yang, Jinhua Zhou, Nicholas Jennings, Cristian Rodriguez-Aguayo, Gabriel Lopez-Berestein, Roberto Miranda, Wei Qiao, Veera Baladandayuthapani, Zongfang Li, Anil K. Sood, Jinsong Liu, Xiao-Feng Le, Robert C. Bast

**Affiliations:** 1 Departments of Experimental Therapeutics, University of Texas MD Anderson Cancer Center, Houston, Texas, United States of America; 2 Department of General Surgery, the Second Affiliated Hospital, School of Medicine, Xi’an Jiaotong University, Xi’an, Shaanxi, China; 3 Departments of Pathology, University of Texas MD Anderson Cancer Center, Houston, Texas, Untied States of America; 4 Bioinformatics Computer Biology, University of Texas MD Anderson Cancer Center, Houston, Texas, United States of America; 5 Departments of Gynecologic Oncology, University of Texas MD Anderson Cancer Center, Houston, Texas, United States of America; 6 Center for RNA Interference and Non-Coding RNA, University of Texas MD Anderson Cancer Center, Houston, Texas, United States of America; University of Hawaii Cancer Center, UNITED STATES

## Abstract

Cyclin-dependent kinase 5 (CDK5) is a cytoplasmic serine/ threonine kinase. Knockdown of CDK5 enhances paclitaxel sensitivity in human ovarian cancer cells. This study explores the mechanisms by which CDK5 regulates paclitaxel sensitivity in human ovarian cancers. Multiple ovarian cancer cell lines and xenografts were treated with CDK5 small interfering RNA (siRNA) with or without paclitaxel to examine the effect on cancer cell viability, cell cycle arrest and tumor growth. CDK5 protein was measured by immunohistochemical staining of an ovarian cancer tissue microarray to correlate CDK5 expression with overall patient survival. Knockdown of CDK5 with siRNAs inhibits activation of AKT which significantly correlates with decreased cell growth and enhanced paclitaxel sensitivity in ovarian cancer cell lines. In addition, CDK5 knockdown alone and in combination with paclitaxel induced G1 cell cycle arrest and caspase 3 dependent apoptotic cell death associated with post-translational upregulation and nuclear translocation of TP53 and p27^Kip1^ as well as TP53-dependent transcriptional induction of p21^Cip1^ in wild type TP53 cancer cells. Treatment of HEYA8 and A2780 wild type TP53 xenografts in nu/nu mice with CDK5 siRNA and paclitaxel produced significantly greater growth inhibition than either treatment alone. Increased expression of CDK5 in human ovarian cancers correlates inversely with overall survival. CDK5 modulates paclitaxel sensitivity by regulating AKT activation, the cell cycle and caspase-dependent apoptosis. CDK5 inhibition can potentiate paclitaxel activity in human ovarian cancer cells.

## Introduction

In the United States in 2014 there were approximately 21,980 new cases of ovarian cancer and 14,270 deaths from this disease, consistent with a cure rate of only 30% for all stages. Improved outcomes might be attained if sensitivity to primary chemotherapy were enhanced. Two major types of epithelial ovarian cancer have been identified. Type I “low grade” cancers grow slowly and are often detected in early stage. At a molecular level, Type I cancers have wild type *TP53* and are driven by activating mutations in Ras and different members of the PI3K signaling pathway. Type II “high grade” cancers grow more rapidly and are often diagnosed in advanced stage. High grade ovarian cancers exhibit mutated *TP53* as well as frequent abnormalities in homologous recombination repair of DNA and are driven by numerous DNA copy number abnormalities, but only very rarely by activating mutations. Both types of ovarian cancer are treated with cytoreductive surgery and a combination of drugs that includes carboplatin and paclitaxel. To enhance the efficacy of paclitaxel for treatment of ovarian cancer, we performed a kinome siRNA library screen in the presence and absence of paclitaxel to identify kinases that regulate paclitaxel sensitivity. Knockdown of CDK5 enhanced paclitaxel sensitivity [1]. CDK5 is required for proper neuronal migration, synapse formation, and survival. Hyperactivation of CDK5 is connected with severe neurodegenerative disorders, including Alzheimer’s disease [2–5]. Recently, dysregulation of CDK5 has been linked to malignancy, including cancers of the prostate, pancreas, thyroid, lung, cervix, myeloma, and breast [6–13].

In this study, we have found that CDK5 knockdown inhibits phosphorylation of AKT, and induces G1 cell cycle arrest, apoptosis and enhanced sensitivity to paclitaxel in ovarian cancer cell lines. In addition, induction of G1 arrest and apoptosis by CDK5 knockdown relates to induction of TP53, p21^Cip1^ and p27^Kip1^ protein. CDK5 inhibition provides a novel strategy for managing ovarian cancers with and without wild-type TP53 function.

## Materials and Methods

### Cell lines and cultures

HEY, A2780, CAOV3, ES-2 and SKOv3 human ovarian cancer cell lines were purchased from the American Type Culture Collection (Manassas, VA). EF021, EF027, OAW42, OC316 and IGROV1 were kindly provided by Dr. Gordon Mills’ laboratory [14–17] and all the cell lines were confirmed with STR DNA fingerprinting which was done by the MDACC Characterized Cell Line core (supported by NCI # CA016672). SKOv3 cells were culture with Macoy’s 5A; OC316, EFO27, EFO21, IGROV1, ES-2, A2780 and Hey cells were culture with RPMI1640; CAOV3 and OAW42 cells were cultured with DMEM. All media were obtained from the Media Preparation Core Facility at M. D. Anderson Cancer Center. SW626 cells were cultured with Leibovitz’s L-15 (Sigma-Aldrich, St. Louis, MO). All cell lines were tested for mycoplasma with a MycoSensor PCR Assay Kit from Stratagene (La Jolla, CA) and found to be free from contamination.

### siRNA and plasmid transfection

All the cell lines were transfected with a negative control siRNA or a specific siRNA using DharmaFECT 4 reagent (GE Dharmacon, Lafayette, CO). A mixture of siRNA (15 nM final concentration) and DharmaFECT 4 reagent (12.5 nM final concentration) was incubated for 20 minat room temperate before being applied to the cells.

### Cell growth assays

A crystal violet assay was used to assess anchorage-dependent cell growth as described previously. Briefly, HEY (6 × 10^3^) or A2780 (8 × 10^3^) cells were seeded in triplicate in 96-well cell culture plates and either reverse transfected for 24 hours with a negative control siRNA or a CDK5 siRNA and incubated for an additional 48 hours with or without paclitaxel (3 nM). The cells were washed with PBS, fixed in 1% glutaraldehyde, and stained with 0.5% crystal violet (Sigma-Aldrich, St. Louis, MO) dissolved in methanol. The dye that stained the cells on the plates was eluted with Sorenson buffer (0.9% sodium citrate, 0.02 N HCl, and 45% ethanol) and directly measured with the use of a Vmax microplate reader (Molecular Devices, Sunnyvale, CA) at a wavelength of 570 nm.

### Clonogenic assays

A clonogenic assay was performed as described previously [18]. Briefly, HEY or A2780 cells were seeded at 500 and 800 cells per well in triplicate after transfected for 24 h with a negative control siRNA or a CDK5 siRNA for 14 days. Cells were then fixed in 1% glutaraldehyde and stained with 0.5% crystal violet in methanol. Colonies with more than 30 cells were counted.

### Flow cytometry

The percentage of cells in the sub-G1 phase of the cell cycle (i.e., apoptotic cells) was determined based on relative DNA concentration measured with a flow cytometry as described previously [1]. Briefly, HEY cells were transfected for 24 h with a negative control siRNA or a CDK5 siRNA and treated with or without paclitaxel (3 nM) for 24 h and were then detached by incubating with 0.05% trypsin-EDTA, washed with PBS, and fixed overnight in 70% ethanol. Fixed cells were then centrifuged, washed, resuspended in PBS containing RNase A and propidium iodide (50 μg/mL each) and incubated for 20 minutes at 37°C with gentle shaking. Stained cells were filtered through nylon mesh (41-μm pore size) and analyzed on a Coulter flow cytometer XL-MCL (Coulter Corporation, Miami, FL). The percentages of sub-G1 population and cell cycle distribution were determined using the MULTICYCLE software program (Phoenix Flow Systems, San Diego, CA).

### qRT-PCR analysis

Cells were harvested and total RNA was extracted from cells using RNA easy kit (Qiagen, Straße 1 Hilden, 40724Germany) according to the manufacturer’s instructions. RNA purity was assessed by measuring absorption at 260 nm (A260) and at 280 nm (A280). Samples that had A260/A280 ratio of 1.9–2.1 were considered acceptable. Integrity of RNA was determined by ethidium bromide staining of 18S and 28S RNA in gels after electrophoresis. RNA concentrations were determined from absorbance at 260 nm (A260). qRT-PCR was performed with the use of a Prism 7900HT Sequence Detection System (Applied Biosystems Incorporated, Foster City, CA) and SYBR Green Universal PCR Master Mix (Bio-Rad Laboratories, Hercules, CA), as described previously [1]. Total RNA isolated from Hey cells transfected with individual siRNAs was reverse transcribed into cDNA with the use of a cDNA kit (Invitrogen, Carlsbad, CA), and the cDNA was subjected to qRT-PCR to assess mRNA levels of p21Cip, TP53, p27Kip1 Bcl2 and CDK5. TP53 (VHPS-9498) and p21Cip (VHPS-1770) primers were purchased from Real Time Primers (Elkins, PA). P27Kip1 primers are 5’-GAGTGGCAAGAGGTGGAGAA-3’ (F) and 5’-GCGTGTCCTCAGAGTTAGCC-3’ (R), Bcl2 primers are 5’-GGAGGATTGTGGCCTTCTTT-3’ (F) and 5’-GCCGTACAGTTCCACAAAGG-3’(R) and CDK5 primers were 5’-ACTCCTACATGGGCAACGAG-3’ (F) and 5’-CGTTCTTCAGGTCGGAGAAG-3’ (R). PCR was performed in a total volume of 30 μL, which included 15 μL of 2X universal PCR master mix, 3 μL of 10× SYBR Green, 20 μM of each forward and reverse primer, and 50 ng of each cDNA sample. Amplifications were carried out in triplicate in 96-well microtiter plates. Thermal cycling conditions were as follows: 95°C for 5 minutes, followed by 40 cycles of 95°C for 10 seconds, and 59°C for 40 seconds. A melting curve analysis was used for all primer sets to exclude nonspecific amplification and consisted of 95°C for 15 seconds, 59°C for 15 seconds, followed by increasing the temperature by 1°C every 2 seconds until 95°C, and then 95°C for 15 seconds.

### Immunoblot and immunoprecipitation

Cells were extracted with lysis buffer (50 mM HEPES, 5 mM EDTA, 100 mM NaCl, 1% Triton X-100 pH 4) containing phosphatase and protease inhibitors. The protein concentration of lysates was determined with a BCA protein assay kit (Pierce, Rockford, IL). Equal amounts of total protein were boiled in sample buffer and separated by SDS-PAGE and transferred onto a polyvinylidene difluoride (PVDF) membrane (Millipore, Bedford, MA). The membranes were incubated with specific antibodies against p-AKT (Cell Signaling, Danvers, MA), p53 (Santa Cruz Biotech, Santa Cruz, CA), p27Kip1 (BD Transduction laboratory, Franklin Lakes, New Jersey), p21Cip (Santa Cruz Biotech, Santa Cruz, CA), caspase 3 (cell signaling, Danvers, MA). Actin or GAPDH (Cell Signaling, Danvers, MA) were used as a loading control in the experiments with cellular proteins. HEY cells (2 × 10^6^) were transfected with either siRNA for 72 h and then lysed in RIPA buffer (20 mM Tris–HCl [pH 7.5], 150 mM NaCl, 1 mM Na2EDTA, 1 mM EGTA, 1% NP-40, 1% sodium deoxycholate, 2.5 mM sodium pyrophosphate, 1 mM b-glycerophosphate, and 1 mM sodium orthovanadate). Briefly, aliquots of the whole-cell lysates (~500 μg) were incubated overnight at 4°C with the mouse anti-CDK5 antibody (~1 μg) (Santa Cruz Biotech, Santa Cruz, CA). Mouse serum was used a negative immunoprecipitation control. Antibody-antigen complexes were collected with the use of protein A-agarose beads (GE Healthcare, Piscataway, NJ) and washed three times washing with RIPA buffer.

### Subcellular fractionation

HEY cells were transfected with negative control and CDK5 siRNA and then fractionated with the use of a Nuclear and Cytoplasmic Extraction Kit (Promega, Madison, WI) according to the manufacturer’s instructions. Equal amounts of protein from the cytoplasmic and nuclear extracts (10 μg) were subjected to immunoblot analysis with antibodies against p21Cip1 (1:300), p27Kip1 (1:1500), PARP (1:1000 dilution; BD, Franklin Lakes, New Jersey), and a-tubulin (1:1000 dilution; Sigma, St. Louis, MO).

### Kinase assays

The in vitro kinase assay was performed by EnzyChrom Kinase Assay Kit (MEDIBENA Life Science & Diagnostic Solutions, Vienna, Austria) according to manufactures’ instruction. Briefly, set up 75 μl reaction mixture containing recombinant CDK5/p25 (Invitrogen) as kinase, ATP and recombinant p53 (Millipore, Billerica, MA), p27Kip1 (Abcam, Cambridge, MA), p21Cip1 (Abcam, Cambridge, MA), Histione H1 (Santa Cruz, Santa Cruz, CA) and ARHI NTD (OriGene, Rockville, MD) as substrate in the Assay Buffer, respectively. Incubate at room temperature for 20 min. Aliquot 20 μl mixture into 3 separate wells on 96 well plate, add 40 μl working reagent to each assay well. Incubate at room temperature for 10 min and read fluorescence intensity (excitation = 530nm and emission = 590nm). Calculate kinase activity as the formula below.

$$Kinase\;activity=\frac{\delta Fluorescence}{slope\times 20min}\times \frac{20\mu l}{75\mu l}\;\left(\frac{unit}{L}\right)$$

δFluorescence = (fluorescence intensity of sample well—blank well) and slope is the slope of the ADP standard curve.

### Luciferase promoter assays

Luciferase activity assays were performed as following. Cells were seeded in 6-well plates, cotransfected with siRNA or its negative control and Renilla luciferase vector (pRL-TK) served as an internal control. After transfection for 16 h, cells were split into 12-well plates, harvested after 24 h and Firefly and Renilla luciferase activities were measured sequentially using the dual luciferase assay kit (Promega, Madison, WI) and a luminometer (BD Parmingene, Sparks, MD). WWP-Luc 9p21/WAF1 promoter was purchased from Addgene (16451, Cambridge, MA). Results were expressed as relative luciferase activity after normalization with Renilla luciferase activity. Relative luminescence units (RLU) were normalized with protein concentrations in each sample, and final values of RLU were expressed as RLU per micro-gram of protein per milliliter.

### Apoptosis blocking with caspase Inhibiton

HEY cells were treated for 2 h with 20 μM of a pan-caspase inhibitor (Z-VAD-FMK, Promega, Madison, WI), or a negative control (Z-FA-FMK, Promega, Madison, WI), after transfected with a negative control siRNA or a CDK5 siRNA for 24 h. The cells were then fixed in 70% ethanol, stained with propidium iodide, and subjected to analysis by flow cytometry. The sub-G1 cell fraction is considered the apoptotic cell population.

### Protein half-life assay

HEY cells were transfected with a negative control siRNA or a CDK5 siRNA for 24 h, washed, and treated with 5 μg/mL of cycloheximide (CHX) for the indicated times. The cells were harvested and use to prepared whole-cell lysates, which were subjected to immunoblot analysis with antibodies against p27Kip1 and glyceraldehyde 3-phosphate dehydrogenase (GAPDH).

### Human ovarian cancer xenografts in nude mice

Experiments with Nu/Nu mice were reviewed and approved by the Institutional Animal Care and Use Committee (IACUC ID: 00001052-RN00) (M. D. Anderson Cancer Center). 60 female nude mice were injected intraperitoneally (i.p.) with 1x106 A2780 cells. At day 7 after injection, the mice were randomly assigned to the following treatment groups (n = 10 mice per group for each cell line): 1) i.p. injection with PBS (twice per week, 100ul/mouse), 2) i.p. injection with paclitaxel (once per week, 30ug/mouse), 3) i.p. injection with control siRNA-DOPC (twice per week), 4) i.p. injection of CDK5 siRNA-DOPC (twice per week, 5ug/mouse), 5) i.p. injection with a combination of control siRNA-DOPC (twice per week) plus paclitaxel (once per week, 30 ug/mouse), and 6) i.p. injection with a combination of CDK5 siRNA-DOPC (twice per week, 5 ug/mouse) plus paclitaxel (once per week, 30 ug/mouse). All mice were treated for 3 weeks and sacrificed by CO2 and cervical dislocation at the end of the studies. All tumors were collected immediately after death and weighed.

### Immunohistochemistry

A formalin-fixed, paraffin embedded tissue microarray (TMA) samples of primary ovarian cancers (215 patient samples) was obtained from the MD Anderson Pathology Department. The protocols for handling paraffin-embedded ovarian cancer specimens and analyzing patient data were approved by the ethical committees of the MDACC Institutional Review Boards. Specimens and associated clinical information were collected under written informed consents, which were signed by each enrolled patient, under the ethics guidelines and approval of the MDACC Institutional Review Boards. For this study, tissue microarray construction was performed as previously described [19]. Tissue microarray slides were subjected to immunohistochemical staining according to the manufacturer’s protocol (Biocare Medical, Concord, CA, USA). Six micron sections were cut from each TMA. Incubation at 60°C for 20 min was used to restore antigenic reactivity followed by two 20 min incubations in Xylene. After slides were rehydrated, antigen retrieval was performed in 6.5 mM sodium citrate buffer (pH 6.0) for 10 min. 3% bovine serum albumin in 0.1 M Tris-buffered saline was used for blocking. Sections were incubated at 4°C overnight with anti-CDK5 antibody (1:100, Sigma HPA018977). Anti-rabbit immunoglobulin secondary antibodies were then applied for 1 h at room temperature followed by washing 3 times in PBS for 10 min. DAB chromagen was added for 1 min per slide followed by 3 additional washes in PBS for 10 min and then hematoxylin staining was performed for 1 min per slide followed by 3 additional washes in PBS for 10 min. Two gynecologic pathologists (J.L, R.M.) independently analyzed the immuno-histochemically stained slides for CDK5 expression.

### Statistical analysis

Data are represented as means +/- standard deviations unless specified otherwise. Statistical significance was determined by independent sample Mann-Whitney U test. Survival analysis was performed by the Kaplan-Meier method. The minimal level of significance was P = 0.05.

## Results

### CDK5 knockdown inhibits cell growth and increases paclitaxel sensitivity in ovarian cancer cells

**Fig 1:**
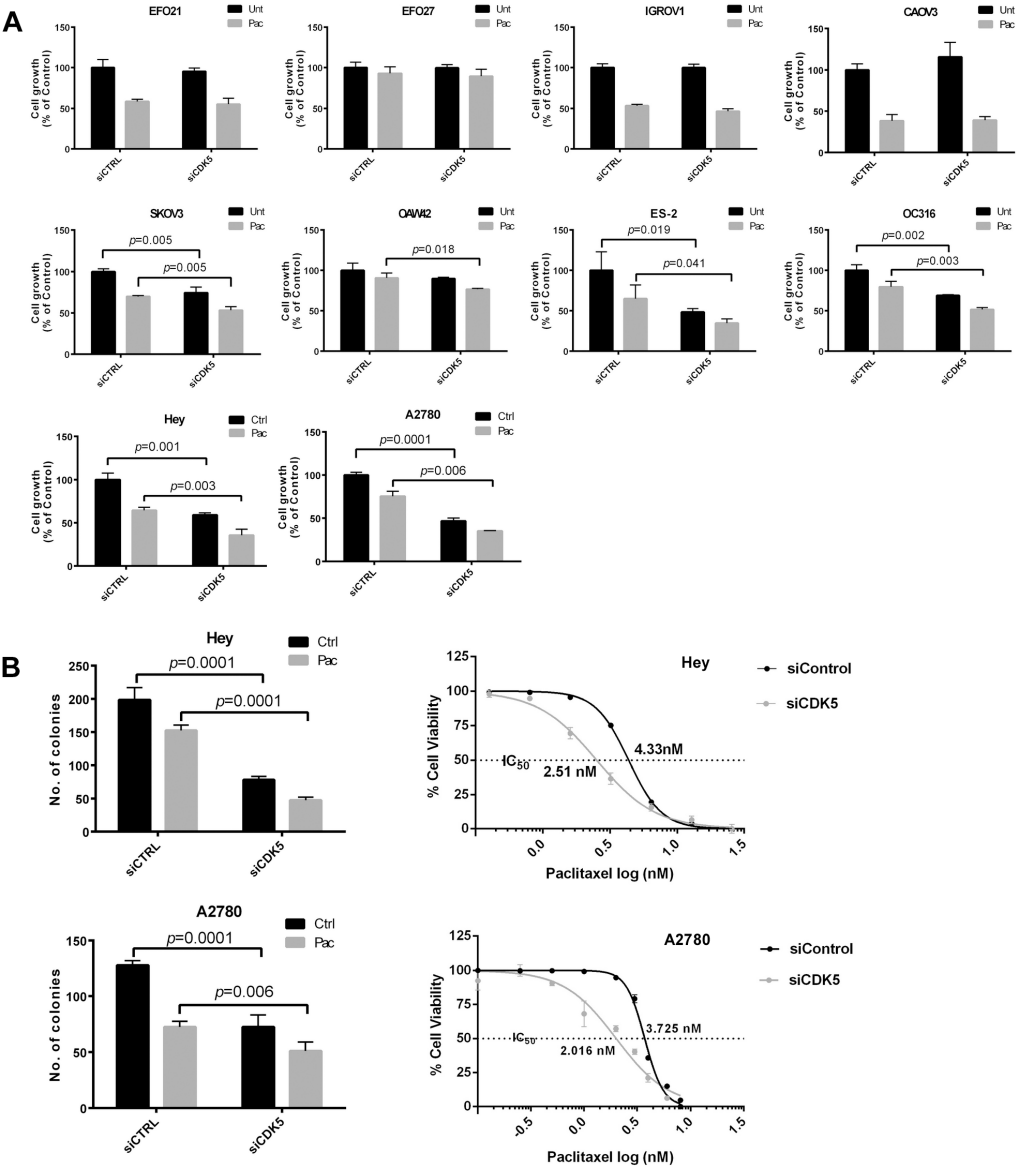
CDK5 siRNA inhibits cell growth and enhances paclitaxel sensitivity in multiple ovarian cancer cell lines. (**A**) Short term cell growth assay. Hey and A2780 cells in 96-well plates were reverse transfected with control siRNA or CDK5 siRNA (Dhamarcon) for 24 h and treated with 5 nM paclitaxel (Pac) or diluent (Control) for an additional 48 h. A crystal violet cell proliferation assay was used to measure the growth of cells. (**B**) Clonogenic and cell growth assays. HEY and A2780 cells were transfected with control siRNA or CDK5 siRNA for 24 h, and then cells were treated with diluent (Control) or 3 nM paclitaxel (Pac) for 14 days (clonogenic assay) or cells were treated with multiple concentrations of paclitaxel (Pac) or diluent (Control) for an additional 48 h (cell growth assays). The IC50s of paclitaxel with or with CDK5 siRNA treatment in Hey and A2780 cells were calculated in GraphPad.

While knockdown of CDK5 with siRNA could alter cell growth and paclitaxel sensitivity in ovarian cancer cell lines with mutant TP53 (EFO21, EFO27, IGROV1, CAOV3, OAW42 and ES-2, SKOv3 and OC316) (Fig 1A), the most marked effects were observed in two ovarian cancer cell lines with wild type TP53 (HEY and A2780) (Fig 1A and 1B). Silencing CDK5 significantly reduced the IC50 of paclitaxel in Hey and A2780 cells (Fig 1B). Forced expression of CDK5 enhanced growth and decreased sensitivity to paclitaxel in HEY cells (S1 Fig). The CDK inhibitor roscovitine also inhibited CDK5 activity and cell growth in Hey cells as did CDK5 siRNA in HEY and A2780 cells (S1 Fig). In addition to inhibiting growth in short term assays, knockdown of CDK5 significantly inhibited colony formation and enhanced sensitivity to paclitaxel in HEY and A2780 cells (Fig 1B and S2 Fig).

### CDK5 knockdown inhibits the activation of AKT

**Fig 2:**
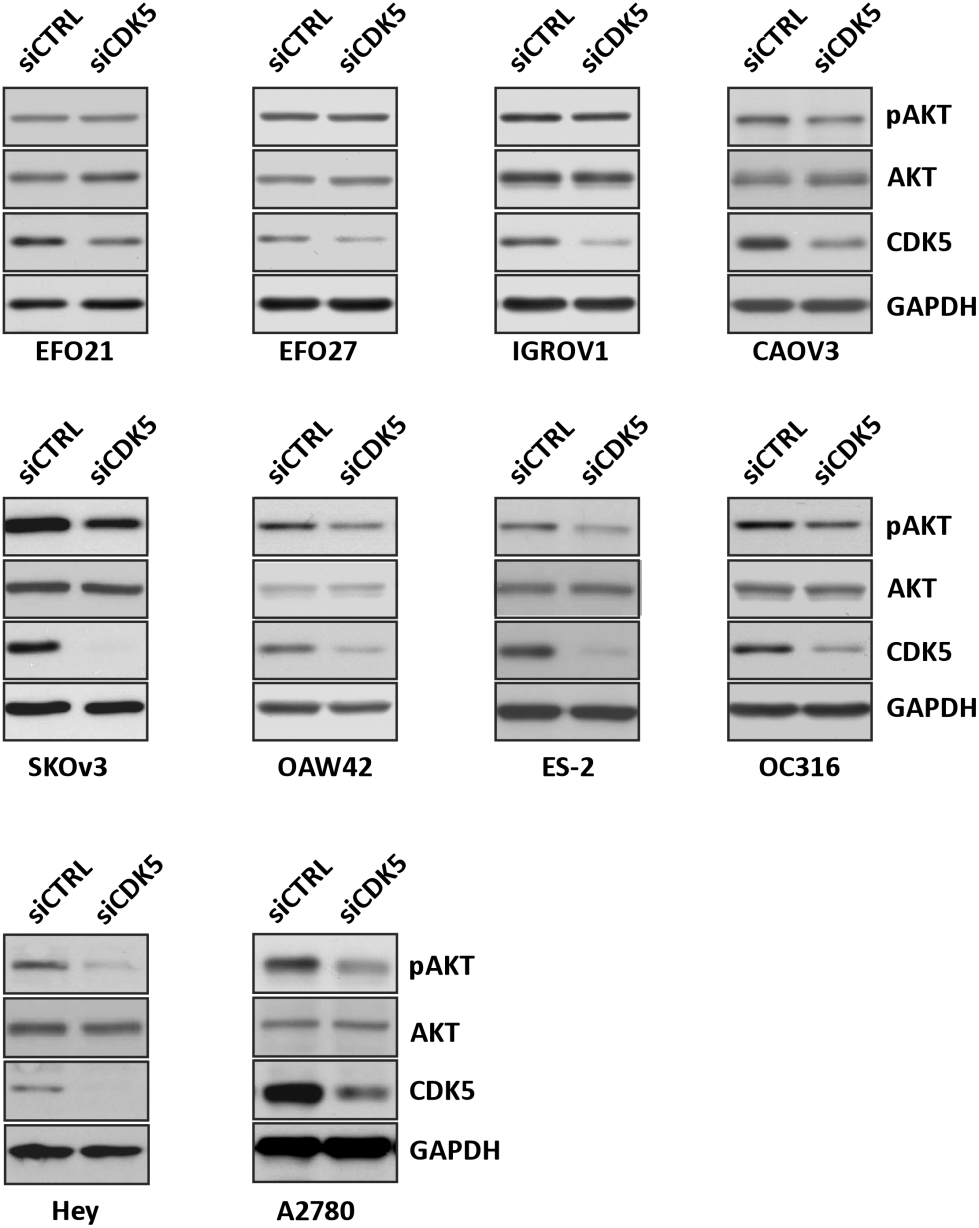
Knockdown of CDK5 inhibits activation of AKT in cancer cell lines. Western analysis for measuring inhibition of AKT phosphorylation (Ser473) using specific anti-p-AKT antibody. HEY cells were treated with control siRNA or CDK5 siRNA for 72 h. Cell lysates was subjected to immunoblot analysis.

The phosphatidyinositol-3-kinase (PI3K)-Akt survival cascade is thought to be associated with the sensitivity to anticancer drugs. To explore the mechanism by which CDK5 silencing inhibited ovarian cancer cell growth and enhanced paclitaxel sensitivity, we first examined the effect of CDK5 knockdown on activation of AKT by detecting Ser473 phosphorylation of AKT. After treatment with CDK5 siRNA or control siRNA, Western analysis was performed. Silencing CDK5 significantly inhibited activation of AKT in 2 wild type TP53 (Fig 2) as well as in 4 of 8 mutant TP53 ovarian cancer cell lines (Fig 2). siRNAs inhibition of AKT activation in 6 ovarian cancer lines significantly correlates with decreased cell growth and enhanced paclitaxel sensitivity (Figs 1A and 2).

### CDK5 knockdown increases apoptosis and G1 cell cycle arrest in the presence and absence of paclitaxel

**Fig 3:**
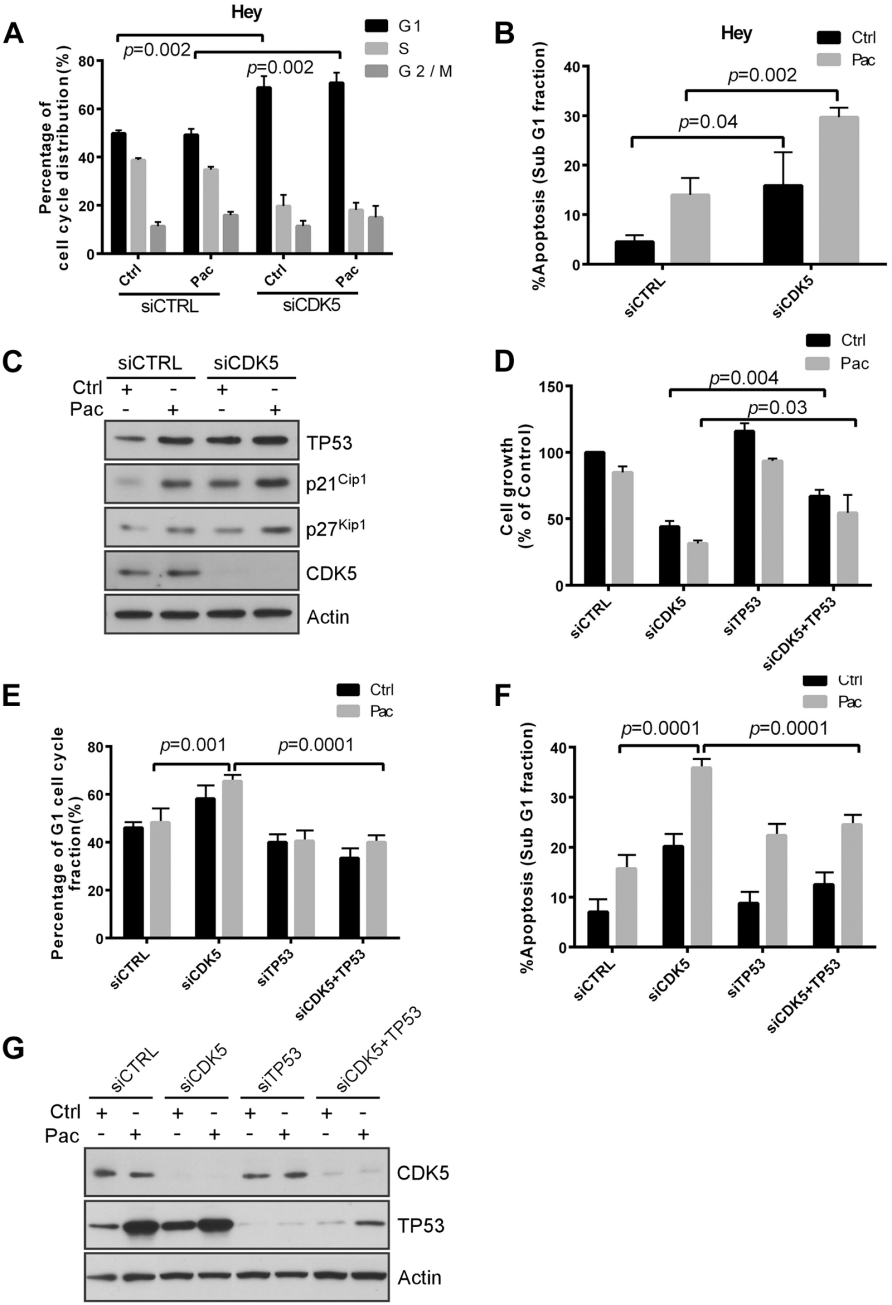
CDK5 knockdown induces p53-dependent growth inhibition, apoptosis and G1 arrest. (**A, B**) Knockdown of p53 reduced CDK5 knockdown-induced apoptosis (A) and G1 arrest (B). HEY cells were reverse transfected with control siRNA or CDK5 siRNA for 24 h and treated with 5 nM paclitaxel (Pac) or diluent (Control) for an additional 48 h, and then cell cycle analyzed by flow cytometry. Data shown are mean values from three independent experiments. (**C**) CDK5 knockdown increased the expression of p21^Cip1^, p53, and p27^Kip1^. HEY cells were treated with control siRNA or CDK5 siRNA for 24 h and then with paclitaxel (3 nM) or diluent for 48 h. Immunoblot analysis was performed with antibodies against p21^Cip1^, p53, and p27^Kip^. (**D**) Knockdown of p53 expression reduced CDK5 siRNA-induced growth inhibition and reduced the enhancement of paclitaxel sensitivity. Cells were co-transfected with CDK5 siRNA and p53 siRNA for 24 h and treated with paclitaxel (Pac) or diluent. Proliferation of cells was measured with a crystal violet cell proliferation assay. (**E, F**) Knockdown of p53 reduced CDK5 knockdown-induced apoptosis (E) and G1 arrest (F). HEY cells were treated as in (A) and cell cycle analyzed by flow cytometry. Data shown are mean values from three independent experiments. (**G**) Western analysis confirmed increasing of TP53 expression by silencing CDK5. HEY cells were treated with control siRNA or CDK5 siRNA for 24 h and then with paclitaxel (3 nM) or diluent for 48 h. Cells lysates was subjected to immunoblot analysis with p53, CDK5 and actin antibody.

To explore the additional mechanisms by which CDK5 silencing inhibits wild type TP53 ovarian cancer cell growth and enhanced paclitaxel sensitivity, we examined the effect of CDK5 knockdown with or without paclitaxel treatment on induction of apoptosis and cell cycle arrest in Hey cells which is TP53 wild type. After treatment with CDK5 siRNA or control siRNA, flow cytometric analysis was performed. Silencing CDK5 induced G1 arrest and produced apoptotic cell death with an increased fraction of cells in sub-G1 (Fig 3A and 3B). CDK5 silencing also enhanced paclitaxel-induced apoptosis (Fig 3B).

### CDK5 knockdown induces TP53-dependent growth inhibition, apoptosis and G1 arrest

To test the effect of CDK5 silencing on proteins that regulate cell cycle arrest and apoptosis, we performed Western analysis of TP53, p21Cip1 and p27Kip1 after treatment of HEY cells with control siRNA and CDK5 siRNA in the presence and absence of paclitaxel. Knockdown of CDK5 significantly increased TP53, p21^Cip1^ and p27^Kip1^ (Fig 3C). Treatment with paclitaxel further increased levels of these three proteins (Fig 3C). In addition, Knockdown of CDK5 induced growth inhibition and knockdown of TP53 reduced silencing CDK5-mediated growth inhibition in the presence or absence of paclitaxel (Fig 3D). In line with this, the CDK5 siRNA increased cancer cell G1 cell cycle arrest and apoptosis, however knockdown of TP53 reduced the CDK5 siRNA-induced G1 cell cycle arrest in the presence or absence of paclitaxel (Fig 3E and 3G) and decreased the CDK5 siRNA-induced apoptosis (Fig 3F and 3G) in HEY cells. In SKOv3 cells that are TP53 null, knockdown of CDK5 increased the fraction of cells in G2/M after treatment with paclitaxel (S3 Fig). Forced expression of wild type TP53 in SKOv3 cells increased the fraction of cells in G1 (S3 Fig).

### CDK5 knockdown produces post-translational upregulation and nuclear translocation of TP53 and p27^Kip1^ and induces TP53-dependent transcription of p21^Cip1^

**Fig 4:**
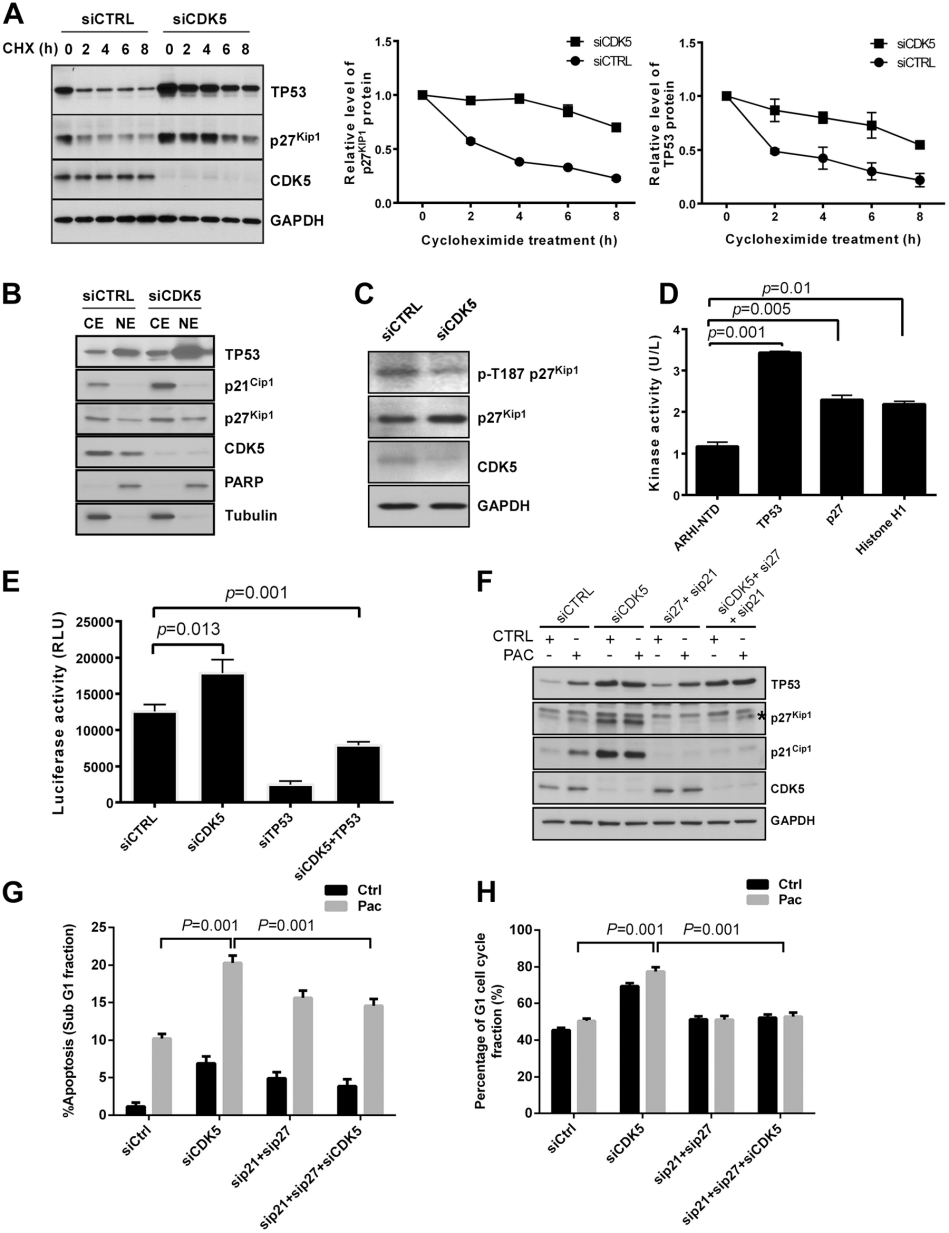
CDK5 knockdown increases TP53 and p27^Kip1^ protein half-lives, increases nuclear localization of the two proteins, decreases p27^Kip1^ p-T187 phosphorylation and induces p21^Cip1^ in a TP53-dependent manner. (**A**) Transfection with CDK5 siRNA increased the half-life of p53 and p27^Kip1^ protein. HEY cells were transfected with control siRNA or CDK5 siRNA for 24 h and then treated with 5 μg/mL of cycloheximide (CHX) for the indicated intervals. Cell lysates was subjected to Western analysis with the antibodies indicated. (**B**) CDK5 siRNA increased the nuclear localization of p53 and p27^Kip1^. HEY cells were transfected with Control siRNA or CDK5 siRNA for 24 h and subjected to fractionation into cytoplasmic and nuclear components. Cytoplasmic (CE) and nuclear (NE) extracts were analyzed by immunoblotting. (**C**) CDK5 siRNA decreased the phosphorylation of p27Kip1 at T187. The cell Immunoblot analysis was performed with antibodies against phosphorylated and total p27Kip1. (**D**) CDK5 enhanced phosphorylation of TP53 and p27^Kip^. A fluorescence based in vitro kinase assay was used to evaluate the interaction of CDK5 with TP53 and p27^Kip1^. (**E**) CDK5 siRNA transcriptionally regulated the expression of p21^Cip1^. HEY cells were transfected with CDK5 siRNA alone or in combination with TP53 siRNA and a luciferase reporter assay was used to measure p21^Cip1^ promoter activity. (**F**) CDK5, p21^Cip1^ and p27^Kip1^ knockdown decreased the expression of CDK5, p21^Cip1^ and p27^Kip1^. HEY cells were treated with control siRNA or CDK5 or/and p21 plus p27 siRNA for 48 h and then with paclitaxel (6 nM) or diluent for 24 h. Immunoblot analysis was performed with antibodies indicated. (**G, H**) Knockdown of p21and p27 reduced CDK5 knockdown-induced apoptosis (G) and G1 arrest (H). HEY cells were treated as in (F) and cell cycle analyzed by flow cytometry after siRNA transfection. SubG1 cells were considered apoptotic cells. Data shown are mean values from three independent experiments.

CDK5 knockdown in HEY cells significantly prolonged the half-life of TP53 and p27^Kip1^ proteins (Fig 4A). Silencing of CDK5 increased nuclear localization of TP53 and p27^Kip1^ proteins and cytoplasmic levels of p21^Cip1^ (Fig 4B). The p27^Kip1^ protein level is primarily regulated post-transcriptionally [20, 21]. CDK5 siRNA decreased the phosphorylation of p27Kip1 at Thr187 (Fig 4C), which is required for binding to ubiquitin ligase and leads to 26S proteasome degradation. TP53 has been reported to be a direct substrate of CDK5 [22–24]. Using purified CDK5 protein and a fluorescence-based in vitro kinase assay, we observed phosphorylation of TP53 and p27^Kip1^ (Fig 4D), using an unrelated protein, ARHI-NTD, as a negative control and Histone H1 as a positive control (S4 Fig) [25, 26]. Because p21^Cip1^ can be regulated by TP53 at the transcriptional level and CDK5 silencing also can upregulate TP53 expression, we tested the effect of CDK5 knockdown and TP53 knockdown on p21^Cip1^ promoter activity. When compared to control siRNA, CDK5 siRNA significantly increased promoter activity of p21^Cip1^, which was abolished by co-transfection with TP53 siRNA (Fig 4E). Furthermore, knockdown of p21^Cip1^ and p27^Kip1^ simultaneously (Fig 4F) reduced the CDK5 siRNA-induced increase in apoptosis (Fig 4G) and decreased the CDK5 siRNA-induced G1 cell cycle arrest in the presence or absence of paclitaxel (Fig 4H) in HEY cells. Thus, silencing of CDK5 produced post-translational upregulation of TP53 and p27^Kip1^ associated with TP53-dependent transcriptional induction of p21^Cip1^.

### CDK5 knockdown-induces apoptosis that depends on caspase-3 activation and is associated with Bcl-2 downregulation

**Fig 5:**
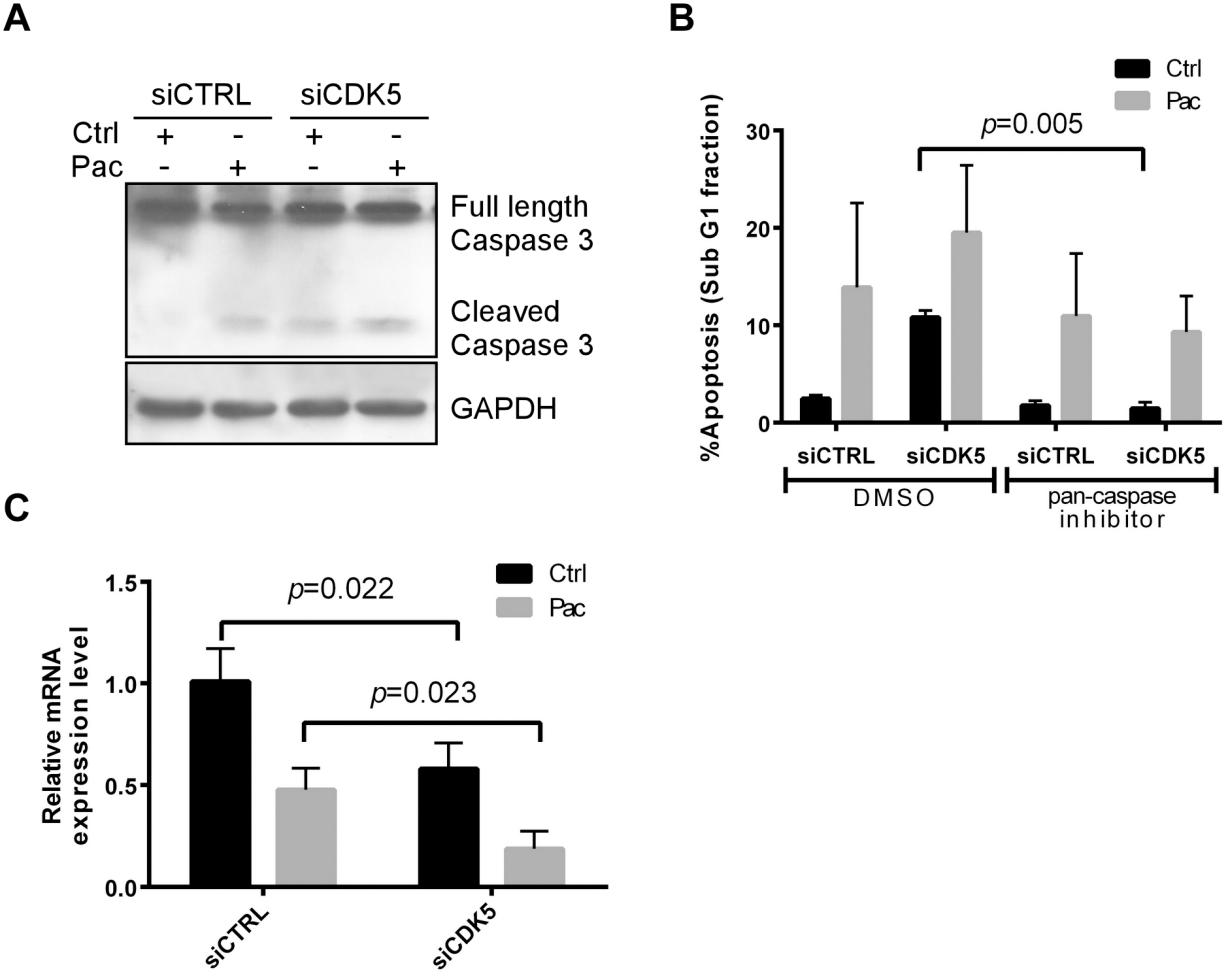
CDK5 knockdown induces caspase-mediated apoptosis and downregulates Bcl-2. (**A**) CDK5 knockdown induced caspase-3 activation in the presence and absence of paclitaxel. HEY cells were transfected with control siRNA or CDK5 siRNA for 24 h, treated with paclitaxel (3 nM) or diluent for 48 h, lysed and total protein analyzed by immunoblot using antibodies against activated caspase-3 and GAPDH. (**B**) A pan-caspase inhibitor blocked CDK5 knockdown–induced apoptosis. HEY cells were pretreated for 2 h with 20 μM of a pan-caspase inhibitor (Z-VAD-FMK), or negative control (Z-FA-FMK), followed by treatment for 24 h with or without paclitaxel (3 nM). The cells were then fixed in 70% ethanol, stained with propidium iodide, and subjected to cell cycle analysis by flow cytometry. The sub-G1 cell population was considered apoptotic and expressed as a percentage of the whole-cell population. (**C**) CDK5 knockdown decreased the expression of Bcl-2 mRNA. HEY cells were treated as in (A) and total RNA was extracted for real-time PCR analysis of Bcl-2 mRNA expression. Data shown were from three independent experiments.

To elucidate the mechanism by which CDK5 knockdown induced apoptosis, we asked whether CDK5 knockdown and paclitaxel treatment activated caspase-3. Western analysis showed that CDK5 knockdown could induce the activation of caspase-3 and this was further increased by treatment with paclitaxel (Fig 5A). Z-VAD-FMK, a pan-caspase inhibitor, blocked apoptosis in cells transfected CDK5 siRNA (Fig 5B). Consequently, the apoptotic cell death induced by CDK5 silencing was caspase dependent. Bcl-2 is an important inhibitor of apoptosis. CDK5 silencing was found to decrease the expression of Bcl-2 mRNA level by qRT-PCR (Fig 5C), which can be accounted for the upregulation of p27Kip1, as we previously reported [1]. The combination of CDK5 siRNA and paclitaxel showed greater inhibition of Bcl-2 mRNA than did CDK5 siRNA alone.

### CDK5 knockdown and treatment with paclitaxel produced additive inhibition of HEYA8 and A2780 human ovarian cancer xenografts

**Fig 6:**
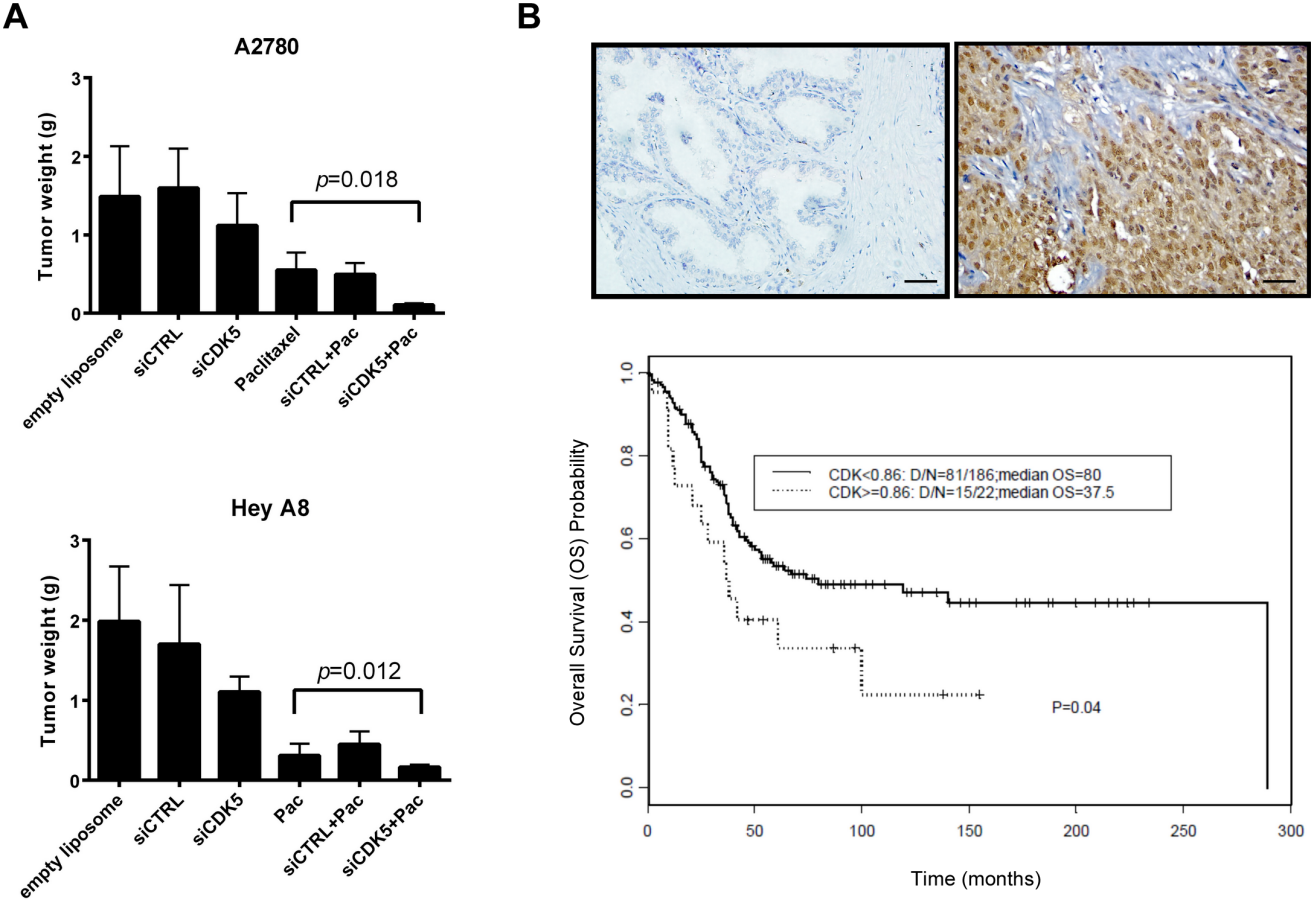
CDK5 siRNA enhances the response to paclitaxel in HEY and A270 human ovarian cancer xenografts and CDK5 expression correlates inversely with ovarian cancer patient survival. (**A**) CDK5 siRNA-DOPC inhibited ovarian cancer xenograft growth and enhanced paclitaxel therapy in nu/nu mice. Groups of 10 mice were injected with HEY A8 or A2780 cancer cells and treated as described in Materials and Methods. Xenograft growth was measured by tumor weight on day 28 after tumor cell injection. (**B**) CDK5 expression correlated with patient survival. TMA Sections (215 patient samples) from department of Pathology were stained with specific antibody against CDK5. Repetitive CDK5 negative and positive images are shown in here. The relation between CDK5 expression level and prognosis was evaluated by Kaplan-Meier survival analysis.

To estimate the potential of CDK5 as a target for clinical therapy, the effect of CDK5 silencing was further tested in the HEYA8 and A2780 orthotopic mouse models of human ovarian cancer in Nu/Nu mice using the well-characterized DOPC (1,2-dioleoyl-sn-glycero-3-phosphatidylcholine) nanoliposomal delivery system [27, 28]. For each cancer cell line, 6 groups of 10 mice were treated for 4 weeks as follows: 1) DOPC nanoliposomes, 2) control siRNA-DOPC, 3) CDK5 siRNA-DOPC, 4) paclitaxel, or 5) a combination of control siRNA-DOPC plus paclitaxel, or 6) a combination of CDK5 siRNA-DOPC plus paclitaxel. CDK5 siRNA-DOPC plus paclitaxel produced greater inhibition of tumor growth than any other group for each of the two models (Fig 6A) and silencing CDK5 decreased p-AKT, increased TP53 and p27^Kip1^ *in vivo* (S5 Fig).

### CDK5 expression correlates inversely with overall survival in ovarian cancer patients

Our observations in cell culture and in mouse models indicated that knockdown of CDK5 inhibited ovarian cancer cell growth and regulated paclitaxel sensitivity. Gene expression array analysis from The Cancer Genome Atlas (TCGA) and from Oncomine (a Cancer Microarray database), suggest that CDK5 mRNA is elevated in at least 30% cases (S6 Fig). When expression of CDK5 was measured by immunohistochemical staining of a tissue microarray with more than 200 cases of primary cancer, we found that patients with high CDK5 expression, particularly expression in the cytoplasm have poor overall survival using Kaplan-Meier survival analysis (Fig 6B and S1 Table). Thus, our data suggest that CDK5 might serve as a prognostic biomarker for ovarian cancer patients.

## Discussion

CDK5 is a ubiquitously expressed proline-directed serine/threonine kinase, active primarily in postmitotic neurons as a result of abundant expression in these cells of its activating partners p35 and/or p39. CDK5 has been considered a neuron-specific kinase and narrowly viewed as an essential regulator of neuronal function. Despite sharing strong sequence identity with other CDK family members, CDK5 has been linked to regulation of the cell cycle only after an interaction was documented between CDK5 and cyclin I [29–31]. Because the neuronal migration during the nervous system development and cancer cell migration and metastasis shared quite similar cellular and molecular mechanisms [32], several studies have linked CDK5 to cancer [33–36]. CDK5 also has been found in the ovary, but only few studies mentioned the role of CDK5 in ovarian cancer [37–39].

The phosphatidyinositol-3-kinase (PI3K)-Akt pathway performs many important functions, including survival [40, 41]. Phosphorylation of PIKE-A by CDK5 mediates growth factor-induced migration and invasion of human glioblastoma cells [42]. PIKE-A exerts its effects by directly regulating Akt, the key downstream effector of the PI3K/PTEN pathway frequently dysregulated in cancer [42]. The PI3K-Akt mediated survival cascade is thought to be associated with the sensitivity to chemotherapy [43, 44]. In this study, knockdown of CDK5 with siRNAs inhibits activation of AKT which significantly correlates with decreased cell growth and enhanced paclitaxel sensitivity in ovarian cancer cell lines. It is possible that CDK5-mediated modulation of AKT may regulate cancer cell survival and chemo-resistant in ovarian cancer.

A link between CDK5 and apoptosis was established by Ahuja et al [45], who confirmed that the high expression in dying cells is unique to CDK5, but not other CDKs (CDK 1–8). The differential increase in CDK5 expression is only at the protein level. They proposed that CDK5 may function in the rearrangement of the cytoskeleton during apoptosis. Recently, Lin et al [46] reported that all-trans retinoic acid could induced growth inhibition in castration-resistant prostate cancer cells through activating CDK5 and p27. Meanwhile, CDK5 inhibition is also connected with increased susceptibility to cell death in many cell types [47, 48]. Wissing et al [49] confirmed that transfection of PC3 prostate cancer cells with a dominant-negative construct CDK5 (PC3 CDK5dn) can enhance inhibition with tilorone on cell growth and invasion. Upadhyay et al [11] reported that carboplatin could induce Cdk5 activation and promotes cell death in breast cancer cell lines.

In this study, we have shown for the first time that knockdown of CDK5 inhibits growth and increases paclitaxel sensitivity of ovarian cancer cell lines and xenografts. Knockdown of CDK5 in TP53 wild type ovarian cancer cells results in increased levels of TP53 with consequent transcriptional induction of p21^Cip1^ and a post-translational increase of p27^Kip1^ that arrests cancer cells in G1 and induces apoptosis that is further enhanced by treatment with paclitaxel. Apoptosis depends upon activation of caspase 3 and is associated with a decrease in levels of the anti-apoptotic protein Bcl-2.

Both TP53 and p27Kip1 have been shown to be substrates for this kinase [22, 50, 51]. In HPV positive cervical cancer, CDK5 can phosphorylate TP53 at Serine20 and Serine46 residues to promote its recruitment on p21^Cip1^ and Bax promoters [22]. During neural stem cell differentiation, CDK5 can phosphorylate p27 at Thr187 and at Ser10, promoting neurite outgrowth as neurons differentiate [50]. In contrast to these reports, this is the first report that associates CDK5 with negative post-translational regulation of TP53 and p27^Kip1^ and transcriptional regulation of p21Cip1 (S7 Fig). Our data suggest that the role of CDK5 is highly context dependent.

Small molecule inhibitors of CDKs are currently being developed as anticancer drugs based on their anti-proliferative activity. Pharmacological and genetic inhibition of CDK5 has inhibited growth of breast and thyroid cancer cell lines [11, 52]. Our data document that knockdown of CDK5 with siRNA can induce apoptosis and G1 arrest in ovarian cancer cell lines and xenografts. Roscovitine can inhibit CDK5 and block growth of several ovarian cancer cell lines. The role of CDK5 in combination with chemotherapy is, however, contextual. In breast cancer cell lines treated with carboplatin, increased CDK5 activity can stimulate p53 stability and lead to cell death. Conversely, decreased expression of CDK5 has seen in the ovarian cancer cells treated with a curcumin analog that can re-sensitize cisplatin-resistant ovarian cancer [53].

In our current study, primary sensitivity to paclitaxel has been enhanced by depletion of CDK5 in cells without acquired resistance to the agent. As less than half of ovarian cancer patients exhibit primary sensitivity to paclitaxel, there is considerable room for improvement in primary therapy. Inhibition of growth and enhancement of paclitaxel sensitivity was observed in 6 of 10 ovarian cancer cell lines. Responses were observed in the presence of wild type TP53 and in the presence of TP53 mutations, however the greatest activity was observed with wild-type TP53. As specific CDK5 inhibitors become available, trials can be conducted to enhance the initial response of Type I low grade ovarian cancers with wild-type TP53 to paclitaxel therapy.

Resistance to paclitaxel can relate to alterations in cell cycle checkpoint control and to dysregulation of apoptotic mechanisms. Overexpression of Bcl-2 has been shown to contribute to paclitaxel resistance [54]. In our previous study [1], we have confirmed that dasatinib can enhance paclitaxel sensitivity of ovarian cancer cells through p27Kip1-mediated suppression of Bcl-2 expression. In this study, we found that CDK5 knockdown may produce similar alterations in cell signaling. The decrease of phosphorylation of p27Kip1 at Thr187 prolongs the half-life of p27Kip1 protein and leads to the downregulation of Bcl-2 mRNA. CDK5 siRNA and paclitaxel exerted a similar downregulation of Bcl-2 that depended upon prolonged survival of the p27Kip1 protein.

Several studies have demonstrated that CDK5 and its specific activator protein p35 were important for spontaneous metastasis and CDK5/p35 may represent a biomarker for prognosis in patients with prostate and lung cancer [6, 8, 12]. Our data demonstrate that patients with high CDK5 expression, especially the cytoplasmic expression have poor overall survival in ovarian cancer. This maybe can explained by the phenomenon that CDK5 exerts a double protective function in neurons, suppressing the cell cycle in the nucleus and suppressing cell death in the cytoplasm[55].

In summary, CDK5 silencing can induce downregulation of AKT activity, apoptotic cell death and G1 cell cycle arrest in ovarian cancer cells both in cell culture and in xenografts. CDK5 regulates activation of AKT, apoptosis, cell cycle and paclitaxel resistance. Our data support the potential importance of CDK5 as a potential prognostic biomarker and target for molecular therapy.

## Supporting Information

S1 Table MD Anderson ovarian cohort characteristic. (DOCX)

S1 Fig Forced expression of CDK5 increases ovarian cancer cell growth and decreases sensitivity to paclitaxel, whereas the CDK inhibitor roscovitine inhibits growth. (DOCX)

S2 Fig CDK5 siRNA inhibits cell growth in Hey and A2780 ovarian cancer cell lines. (DOCX)

S3 Fig Knockdown of CDK5 increases the fraction of TP53 null SKOv3 ovarian cancer cells in G2/M after treatment with paclitaxel, whereas forced expression of TP53 increases the fraction of SKOv3 cells in G1. (DOCX)

S4 Fig Protein controls for kinase activity assay. (DOCX)

S5 Fig Increased expression of CDK5 mRNA in ovarian cancers; (DOCX)

S6 Fig CDK5 siRNA increased protein level of p53, p27 and inhibited activated AKT in ovarian cancer xenografts. (DOCX)

S7 Fig CDK5 regulates apoptosis and G1 arrest in ovarian cancer cells with wild-type TP53. (DOCX)
