# Supplementary material for: CDK5 Regulates Paclitaxel Sensitivity in Ovarian Cancer Cells by Modulating AKT Activation, p21Cip1- and p27Kip1-Mediated G1 Cell Cycle Arrest and Apoptosis
Source: PLoS One. 2015 Jul 6;10(7):e0131833. doi: 10.1371/journal.pone.0131833 (PMC4492679; doi:10.1371/journal.pone.0131833)
Supplement: S1 Table — (DOCX) [file pone.0131833.s001.docx]

**S8. Table**. **MD Anderson ovarian cohort characteristics**

| **Main_ID** | **Diagnosis** | **Grade** | **Age** | **Relapse** | **Disease free survival** |
| --- | --- | --- | --- | --- | --- |
| 2 | Clear Cell Carcinoma | 3 | 57 | No | 85 |
| 642 | Clear Cell Carcinoma | 3 | 53 | Progressive Disease | 26 |
| 4214 | Clear Cell Carcinoma | 3 | 43 | Yes | 6 |
| 2494 | Clear Cell Carcinoma | 3 | 43 | Progressive Disease | 14 |
| 12 | Clear Cell Carcinoma | 3 | 40 | No | 7 |
| 585 | Endometroid | 3 | 48 | Progressive Disease |  |
| 2311 | Endometroid | 2 | 47 | No | 1 |
| 82 | Endometroid | 2 | 58 | No | 0 |
| 48 | Endometroid | 2 | 47 | No | 0 |
| 4718 | Endometroid | 1 | 53 | No |  |
| 3360 | Endometroid | 3 | 44 | Yes | 2 |
| 1014 | Endometroid | 3 | 76 | No | 33 |
| 2286 | Endometroid | 1 | 33 | No | 6 |
| 75 | Endometroid | 1 | 49 | Yes | 0 |
| 2324 | Endometroid | 1 | 44 | No | 14 |
| 2436 | Endometroid | 3 | 52 | No | 0 |
| 2436 | Endometroid | 3 | 52 | No | 0 |
| 4258 | Endometroid | 3 | 74 | No | 17 |
| 958 | Endometroid | 3 | 71 | N/A | 10 |
| 2285 | Endometroid | 2 | 69 | No | 12 |
| 4330 | Endometroid | 3 | 42 | Yes | 25 |
| 4431 | Endometroid | 3 | 52 | No | 71 |
| 4411 | Endometroid | 3 | 62 | Yes | 43 |
| 146 | Endometroid | 1 | 66 | No | 18 |
| 2318 | Endometroid | 1 | 83 | Progressive Disease |  |
| 914 | Endometroid | 1 | 48 | Yes | 0 |
| 310 | Endometroid | 3 | 72 | Yes | 0 |
| 198 | Endometroid | 2 | 50 | Yes | 3 |
| 4330 | Endometroid | 3 | 42 | Yes | 0 |
| 165 | Endometroid | 2 | 71 | N/A | 26 |
| 164 | Endometroid | 2 | 61 | No |  |
| 211 | Endometroid | 3 | 57 | No | 0 |
| 474 | MMMT | 3 | 49 | Progressive Disease | 0 |
| 4517 | MMMT | 3 | 47 | Yes | 0 |
| 4314 | MMMT | 3 | 63 | Yes | 78 |
| 4241 | MMMT | 3 | 62 | Yes |  |
| 3417 | MMMT | 3 | 67 | Yes | 0 |
| 805 | MMMT | 3 | 61 | Progressive Disease | 0 |
| 4243 | MMMT |  |  |  |  |
| 359 | MMMT | 3 | 59 | Yes | 0 |
| 688 | MMMT | 3 | 67 | N/A | 118 |
| 2296 | Mucinous | 3 | 24 | No | 22 |
| 704 | Mucinous | 4 | 63 | Yes | 13 |
| 2287 | Mucinous | 1 | 47 | No |  |
| 2330 | Mucinous | 3 | 59 | No | 0 |
| 2491 | Mucinous | 2 | 50 | No | 0 |
| 4722 | Mucinous | 3 | 62 | Yes | 0 |
| 711 | Others | 3 | 52 | Progressive Disease | 125 |
| 4498 | Others | 3 | 42 | Yes | 0 |
| 233 | Others | 3 | 68 | Progressive Disease | 13 |
| 854 | Others | 3 | 71 | Progressive Disease |  |
| 4290 | Others | 3 | 51 | Yes | 12 |
| 2582 | Others | 3 | 37 | Yes | 17 |
| 889 | Serous | 3 | 51 | Yes | 0 |
| 1037 | Serous | 3 | 61 | Progressive Disease | 140 |
| 871 | Serous | 3 | 64 | Progressive Disease | 36 |
| 761 | Serous | 3 | 45 | Yes | 0 |
| 637 | Serous | 3 | 50 | N/A | 0 |
| 3414 | Serous | 3 | 59 | Yes | 16 |
| 4387 | Serous | 3 | 55 | Progressive Disease | 11 |
| 2407 | Serous | 3 | 45 | Yes | 0 |
| 355 | Serous | 3 | 60 |  | 117 |
| 885 | Serous | 3 | 73 | No | 0 |
| 588 | Serous | 3 | 61 | Yes | 0 |
| 4359 | Serous | 3 | 64 | Yes | 12 |
| 4597 | Serous | 3 | 53 | Yes | 0 |
| 890 | Serous | 3 | 57 | Yes | 0 |
| 470 | Serous | 3 | 63 | Progressive Disease | 12 |
| 710 | Serous | 3 | 65 | Progressive Disease | 80 |
| 625 | Serous | 3 | 83 | Progressive Disease | 8 |
| 1043 | Serous | 3 | 47 | Yes | 59 |
| 38 | Serous | 3 | 60 | Yes | 0 |
| 4573 | Serous | 3 | 71 | Progressive Disease | 0 |
| 4573 | Serous | 3 | 71 | Progressive Disease | 12 |
| 1047 | Serous | 3 | 60 | Progressive Disease | 14 |
| 4533 | Serous | 3 | 50 | Progressive Disease | 14 |
| 2418 | Serous | 3 |  |  | 0 |
| 4648 | Serous | 3 | 43 | No |  |
| 2398 | Serous | 3 | 64 | Yes | 0 |
| 4744 | Serous | 3 | 45 | Yes | 8 |
| 2407 | Serous | 3 | 45 | Yes | 0 |
| 4387 | Serous | 3 | 55 | Progressive Disease | 0 |
| 446 | Serous | 3 | 77 | Yes | 17 |
| 1030 | Serous | 3 | 63 | Progressive Disease | 0 |
| 4635 | Serous | 3 | 79 | Yes | 49 |
| 4289 | Serous | 3 | 62 |  | 0 |
| 1044 | Serous | 3 | 63 | Progressive Disease | 0 |
| 808 | Serous | 3 | 75 | Progressive Disease | 0 |
| 2545 | Serous | 1 | 63 | Yes | 0 |
| 740 | Serous | 3 | 53 | Progressive Disease | 0 |
| 678 | Serous | 3 | 66 | Progressive Disease | 0 |
| 4323 | Serous | 3 | 61 |  | 9 |
| 527 | Serous | 3 | 53 | Yes |  |
| 4527 | Serous | 3 | 68 | Progressive Disease | 96 |
| 4387 | Serous | 3 | 55 | Progressive Disease | 0 |
| 70 | Serous | 3 | 52 | Yes | 7 |
| 1035 | Serous | 3 | 52 | Yes | 0 |
| 631 | Serous | 3 | 74 | Progressive Disease |  |
| 80 | Serous | 3 | 70 | Progressive Disease |  |
| 4638 | Serous | 3 | 58 | Yes | 13 |
| 850 | Serous | 3 | 73 | Progressive Disease | 6 |
| 700 | Serous | 3 | 61 | Yes | 0 |
| 459 | Serous | 3 | 56 | Progressive Disease | 0 |
| 1049 | Serous | 1 | 45 | Yes | 0 |
| 4276 | Serous | 3 | 63 | Yes | 0 |
| 4268 | Serous | 3 | 54 | Yes | 0 |
| 575 | Serous | 3 | 65 | Progressive Disease | 0 |
| 179 | Serous | 3 | 62 | Progressive Disease | 0 |
| 2418 | Serous | 3 |  |  | 6 |
| 4715 | Serous | 3 | 76 |  | 5 |
| 716 | Serous | 3 | 64 | Yes | 0 |
| 3354 | Serous | 1 | 69 | Progressive Disease | 0 |
| 4396 | Serous | 3 | 50 | Progressive Disease | 0 |
| 469 | Serous | 3 | 73 | Yes | 76 |
| 4530 | Serous | 3 | 47 | Progressive Disease | 0 |
| 4727 | Serous | 3 | 72 | Progressive Disease |  |
| 4257 | Serous | 3 | 61 | Yes | 0 |
| 2440 | Serous | 3 | 71 | Yes | 49 |
| 3412 | Serous | 3 | 65 | Yes | 0 |
| 4544 | Serous | 3 | 51 | Yes | 0 |
| 4717 | Serous | 3 | 54 | Progressive Disease | 0 |
| 2169 | Serous | 3 | 51 | Yes | 0 |
| 622 | Serous | 3 | 63 | N/A | 0 |
| 4276 | Serous | 3 | 63 | Yes | 0 |
| 62 | Serous | 3 | 71 | Yes | 0 |
| 4610 | Serous | 3 | 59 | Yes |  |
| 69 | Serous | 3 | 66 | Yes | 0 |
| 45 | Serous | 3 | 57 | Progressive Disease | 0 |
| 3358 | Serous | 3 | 68 | Progressive Disease | 35 |
| 3352 | Serous | 3 | 54 | N/A | 0 |
| 4709 | Serous | 3 | 60 | Progressive Disease | 0 |
| 4735 | Serous | 3 | 64 | Yes | 0 |
| 4323 | Serous | 3 | 61 |  | 6 |
| 4716 | Serous | 3 | 63 | Progressive Disease | 0 |
| 64 | Serous | 3 | 51 | Progressive Disease | 7 |
| 4400 | Serous | 3 | 75 | Progressive Disease | 0 |
| 322 | Serous | 3 | 48 | Progressive Disease | 0 |
| 4724 | Serous | 1 | 56 | Progressive Disease |  |
| 5 | Serous | 3 | 55 | Progressive Disease | 0 |
| 4729 | Serous | 3 | 64 | Yes |  |
| 341 | Serous | 3 | 66 | N/A | 30 |
| 3355 | Serous | 3 | 89 | No | 0 |
| 4567 | Serous | 3 | 61 | Progressive Disease | 0 |
| 3484 | Serous | 3 | 58 | Progressive Disease | 6 |
| 4519 | Serous | 3 | 45 | No |  |
| 4244 | Serous | 3 | 63 | Yes | 10 |
| 4149 | Serous | 3 | 51 | No | 0 |
| 2575 | Serous | 3 | 72 | Yes | 0 |
| 4641 | Serous | 3 | 58 | Progressive Disease | 0 |
| 4239 | Serous | 3 | 69 | Yes | 0 |
| 4521 | Serous | 3 | 60 | Yes | 0 |
| 4545 | Serous | 3 | 54 |  | 0 |
| 4525 | Serous | 3 | 57 | Yes |  |
| 636 | Serous | 3 | 87 | Yes |  |
| 3340 | Serous | 3 | 75 | Yes | 0 |
| 1023 | Serous | 3 | 34 | Yes | 0 |
| 4380 | Serous | 3 | 73 | Yes | 0 |
| 2546 | Serous | 3 | 57 | Progressive Disease | 79 |
| 4531 | Serous | 3 | 43 | Yes | 10 |
| 4282 | Serous | 3 | 76 | Yes | 0 |
| 3377 | Serous | 3 | 51 |  | 0 |
| 2419 | Serous | 3 | 52 | Yes | 0 |
| 1054 | Serous | 3 | 29 | Yes | 0 |
| 4741 | Serous | 3 | 63 | Yes | 0 |
| 4637 | Serous | 3 | 55 | N/A | 82 |
| 4337 | Serous | 3 | 68 | Progressive Disease |  |
| 4740 | Serous | 3 | 56 | Progressive Disease | 29 |
| 4257 | Serous | 3 | 61 | Yes | 22 |
| 2421 | Serous | 3 | 69 | Progressive Disease | 37 |
| 4259 | Serous | 3 | 50 | Progressive Disease | 0 |
| 4238 | Serous | 3 | 53 | Yes | 0 |
| 4720 | Serous | 3 | 65 | Progressive Disease | 0 |
| 456 | Serous | 3 | 31 | No | 0 |
| 77 | Serous | 3 | 56 | Yes | 0 |
| 3366 | Serous | 3 | 39 | Yes | 0 |
| 4280 | Serous | 3 | 69 | N/A | 0 |
| 4318 | Serous | 3 | 50 | Yes | 19 |
| 4253 | Serous | 3 | 69 | N/A | 0 |
| 842 | Serous | 3 | 65 | No | 0 |
| 2471 | Serous | 3 | 48 | Yes | 50 |
| 241 | Serous | 3 | 61 | Progressive Disease | 0 |
| 218 | Serous | 3 | 64 | Yes | 3 |
| 3359 | Serous | 3 | 62 | N/A | 0 |
| 2695 | Serous | 3 | 53 | Yes | 0 |
| 4602 | Serous | 3 | 75 | Yes | 22 |
| 4302 | Serous | 3 | 48 | No | 16 |
| 4726 | Serous | 3 | 72 | Progressive Disease |  |
| 313 | Serous | 3 | 42 | Yes |  |
| 4238 | Serous | 3 | 53 | Yes | 63 |
| 454 | Serous | 3 | 70 | Yes | 0 |
| 4320 | Serous | 3 | 72 | Yes | 0 |
| 243 | Serous | 3 | 59 | Yes | 15 |
| 139 | Serous | 1 | 21 | Yes | 6 |
| 2425 | Serous | 3 | 58 | Yes | 0 |
| 2415 | Serous | 3 | 72 | Yes | 14 |
| 4713 | Serous | 3 | 68 | Yes | 10 |
| 4240 | Serous | 3 | 68 | Progressive Disease | 0 |
| 190 | Serous | 3 | 71 | Yes | 40 |
| 488 | Serous | 3 | 76 | Progressive Disease | 0 |
| 4711 | Serous | 3 | 54 | Yes | 0 |
| 550 | Serous | 3 | 53 | Progressive Disease | 0 |
| 4599 | Serous | 3 | 60 | Progressive Disease | 0 |
| 4736 | Serous | 3 | 80 | Yes |  |
| 4642 | Serous | 3 | 55 | Progressive Disease | 0 |
| 2429 | Serous | 3 | 45 | Yes | 12 |
| 4737 | Serous | 3 | 39 | Yes | 0 |
| 817 | Serous | 3 | 73 | progressive disease | 0 |
| 4631 | Serous | 3 | 56 |  | 10 |
| 213 | Serous | 3 | 78 | Progressive Disease | 0 |
| 544 | Serous | 3 | 65 | Progressive Disease | 17 |
| 4725 | Serous | 3 | 66 |  | 37 |
| 614 | Serous | 3 | 49 | Yes | 25 |
| 88 | Serous | 3 | 45 | Yes | 38 |
| 97 | Serous | 3 | 46 | Yes | 8 |
| 4654 | Serous | 3 | 54 | Yes | 0 |
| 215 | Serous | 3 | 68 | Progressive Disease | 45 |
| 460 | Serous | 3 | 28 | Progressive Disease | 10 |
| 468 | Serous | 3 | 75 | Progressive Disease | 16 |
| 561 | Serous | 3 | 59 | Progressive Disease | 51 |
| 205 | Serous | 3 | 52 | Progressive Disease | 21 |
| 1001 | Serous | 3 | 79 | Progressive Disease | 2 |
| 757 | Serous | 3 | 65 | Yes |  |
