# Supplementary material for: CDK5 Regulates Paclitaxel Sensitivity in Ovarian Cancer Cells by Modulating AKT Activation, p21Cip1- and p27Kip1-Mediated G1 Cell Cycle Arrest and Apoptosis
Source: PLoS One. 2015 Jul 6;10(7):e0131833. doi: 10.1371/journal.pone.0131833 (PMC4492679; doi:10.1371/journal.pone.0131833)
Supplement: S1 Fig — (DOCX) [file pone.0131833.s002.docx]

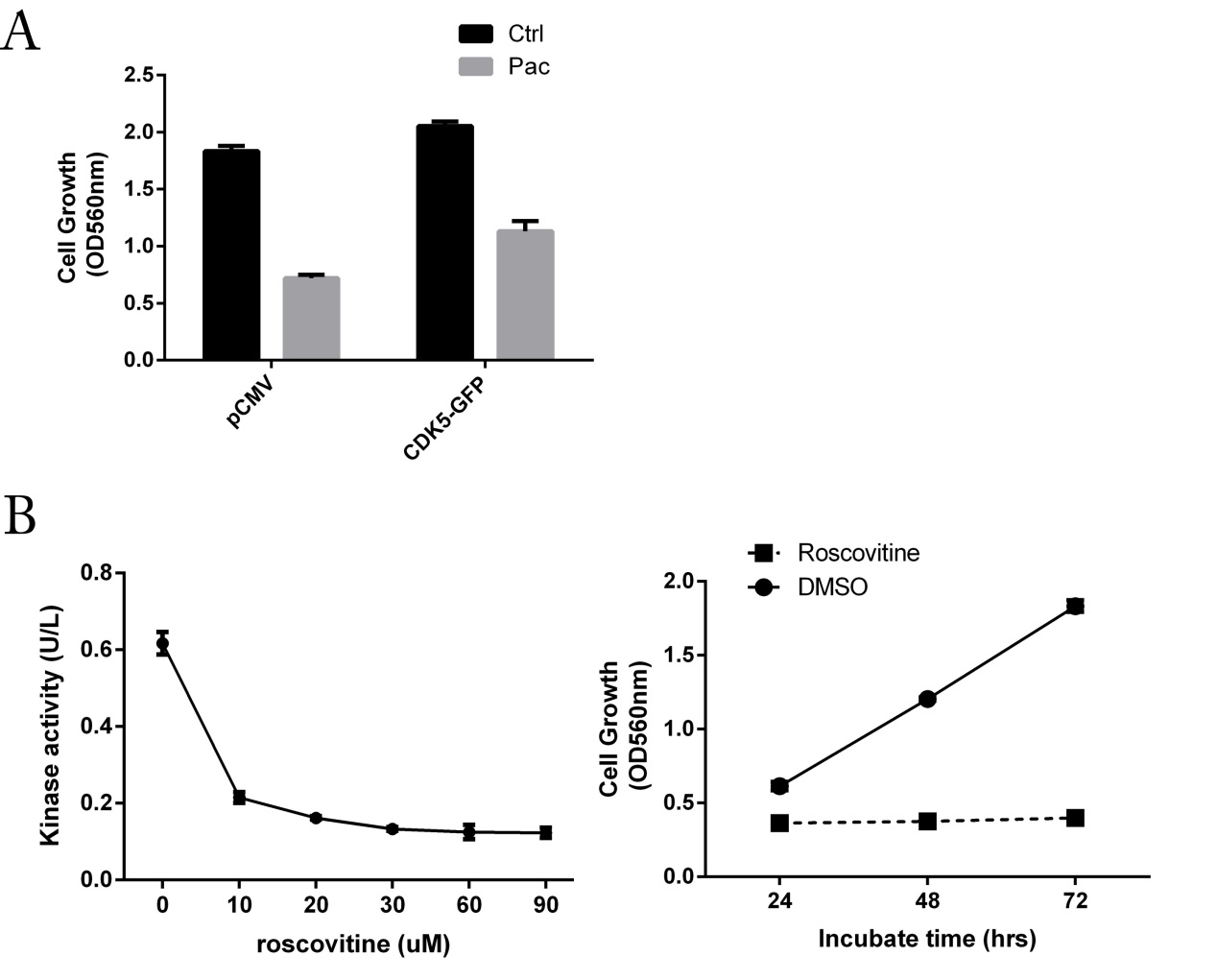


**S1 Fig. Forced expression of CDK5 increases ovarian cancer cell growth and decreases sensitivity to paclitaxel, whereas the CDK inhibitor roscovitine inhibits growth.** (A) Crystal violet growth assay after forced expression of CDK5. HEY cells were transfected with pCMV empty vector or pCMV-CDK5 plasmid for 16hrs and reseeded in 96-well plates prior to a crystal violet cell growth assay was performed after 72 hours. (B) Roscovitine inhibited CDK5 activity (left panel) and inhibited growth of Hey ovarian cancer cell line. Cancer cells were incubated for 24, 48 or 72 hours with rescovitine (30uM) or with diluent prior to a crystal violet cell growth assay.
