# Supplementary material for: CDK5 Regulates Paclitaxel Sensitivity in Ovarian Cancer Cells by Modulating AKT Activation, p21Cip1- and p27Kip1-Mediated G1 Cell Cycle Arrest and Apoptosis
Source: PLoS One. 2015 Jul 6;10(7):e0131833. doi: 10.1371/journal.pone.0131833 (PMC4492679; doi:10.1371/journal.pone.0131833)
Supplement: S2 Fig — (DOCX) [file pone.0131833.s003.docx]

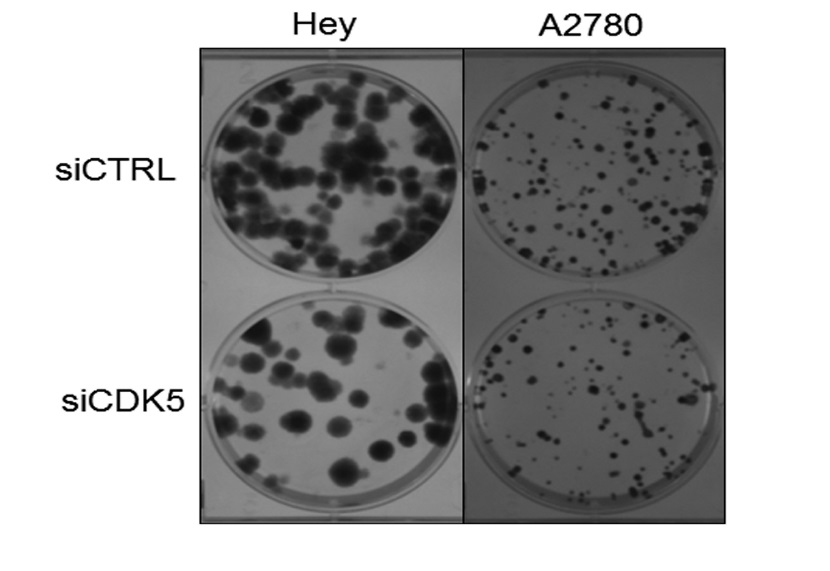


**S2 Fig. CDK5 siRNA inhibits cell growth in Hey and A2780 ovarian cancer cell lines**. A crystal violet cell growth assay was used to measure growth in Hey and A2780 cell lines. Cells were transfected with control siRNA or CDK5 siRNA for 24 hours prior to treatment with paclitaxel (3 nM) or diluent for 48 hrs, and then cells were continually incubated for 14 days.
