# Supplementary material for: CDK5 Regulates Paclitaxel Sensitivity in Ovarian Cancer Cells by Modulating AKT Activation, p21Cip1- and p27Kip1-Mediated G1 Cell Cycle Arrest and Apoptosis
Source: PLoS One. 2015 Jul 6;10(7):e0131833. doi: 10.1371/journal.pone.0131833 (PMC4492679; doi:10.1371/journal.pone.0131833)
Supplement: S3 Fig — (DOCX) [file pone.0131833.s004.docx]

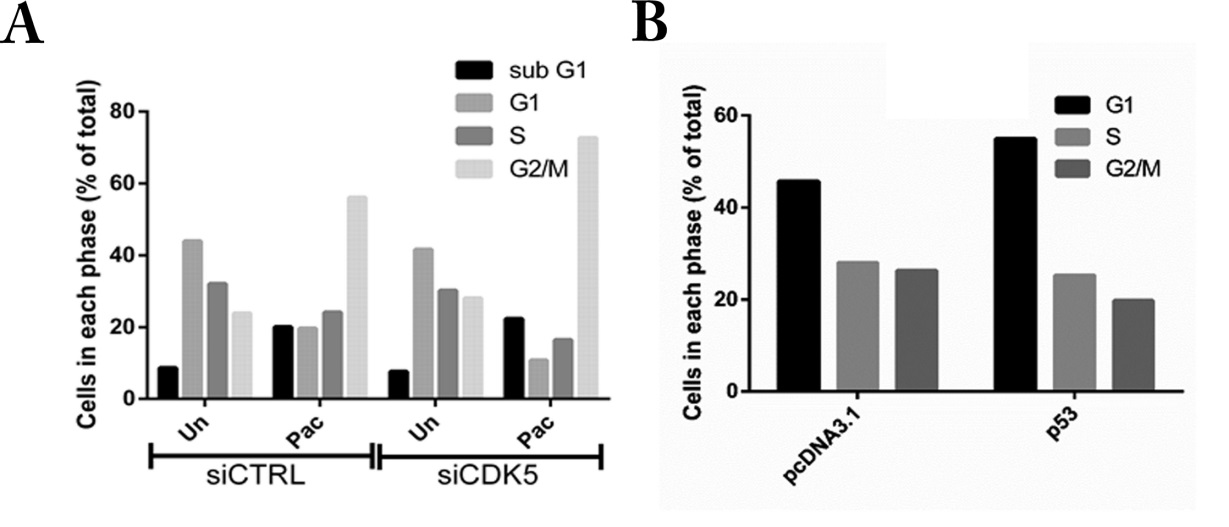


**S3 Fig. Knockdown of CDK5 increases the fraction of TP53 null SKOv3 ovarian cancer cells in G2/M after treatment with paclitaxel, whereas forced expression of TP53 increases the fraction of SKOv3 cells in G1.** (A) SKOv3 cells were transfected with control siRNA or CDK5 siRNA for 24 hours and treated with paclitaxel (3 uM) for 48 hours before analysis with flow cytometry. Values represent the mean and standard deviation of 2 independent experiments. (B) SKOv3 cells were transfected with a pcDNA3.1 vector or a pcDNA-p53 plasmid. After 24 hours cell cycle analysis was performed by flow cytometry.
