# Supplementary material for: CDK5 Regulates Paclitaxel Sensitivity in Ovarian Cancer Cells by Modulating AKT Activation, p21Cip1- and p27Kip1-Mediated G1 Cell Cycle Arrest and Apoptosis
Source: PLoS One. 2015 Jul 6;10(7):e0131833. doi: 10.1371/journal.pone.0131833 (PMC4492679; doi:10.1371/journal.pone.0131833)
Supplement: S4 Fig — (DOCX) [file pone.0131833.s005.docx]

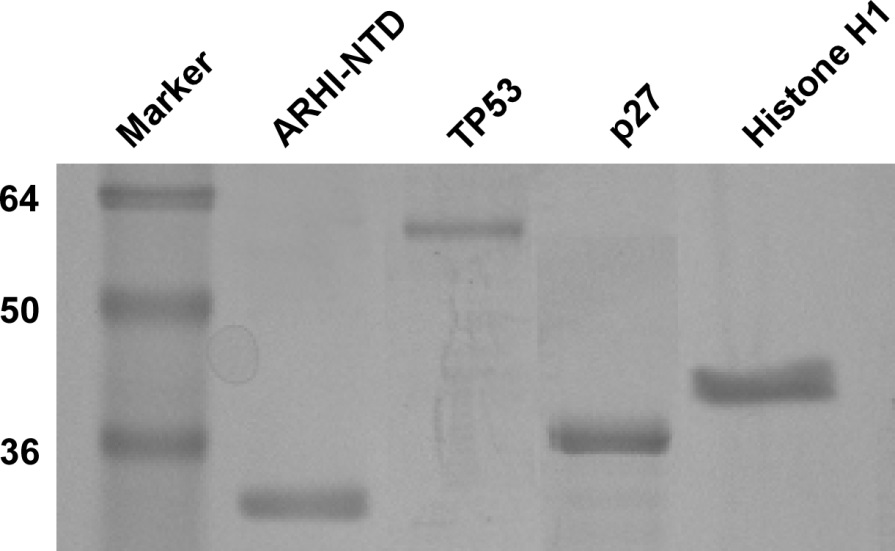


**S4 Fig**. **Protein controls for kinase activity assay**. ARHI-NTD, TP53, p27 and Histone H1 fusion proteins was separated in a SDS page gel (1 ug/ml of protein in each lane) and coomassie blue was used to stain the gel.
