# Supplementary material for: CDK5 Regulates Paclitaxel Sensitivity in Ovarian Cancer Cells by Modulating AKT Activation, p21Cip1- and p27Kip1-Mediated G1 Cell Cycle Arrest and Apoptosis
Source: PLoS One. 2015 Jul 6;10(7):e0131833. doi: 10.1371/journal.pone.0131833 (PMC4492679; doi:10.1371/journal.pone.0131833)
Supplement: S5 Fig — (DOCX) [file pone.0131833.s006.docx]

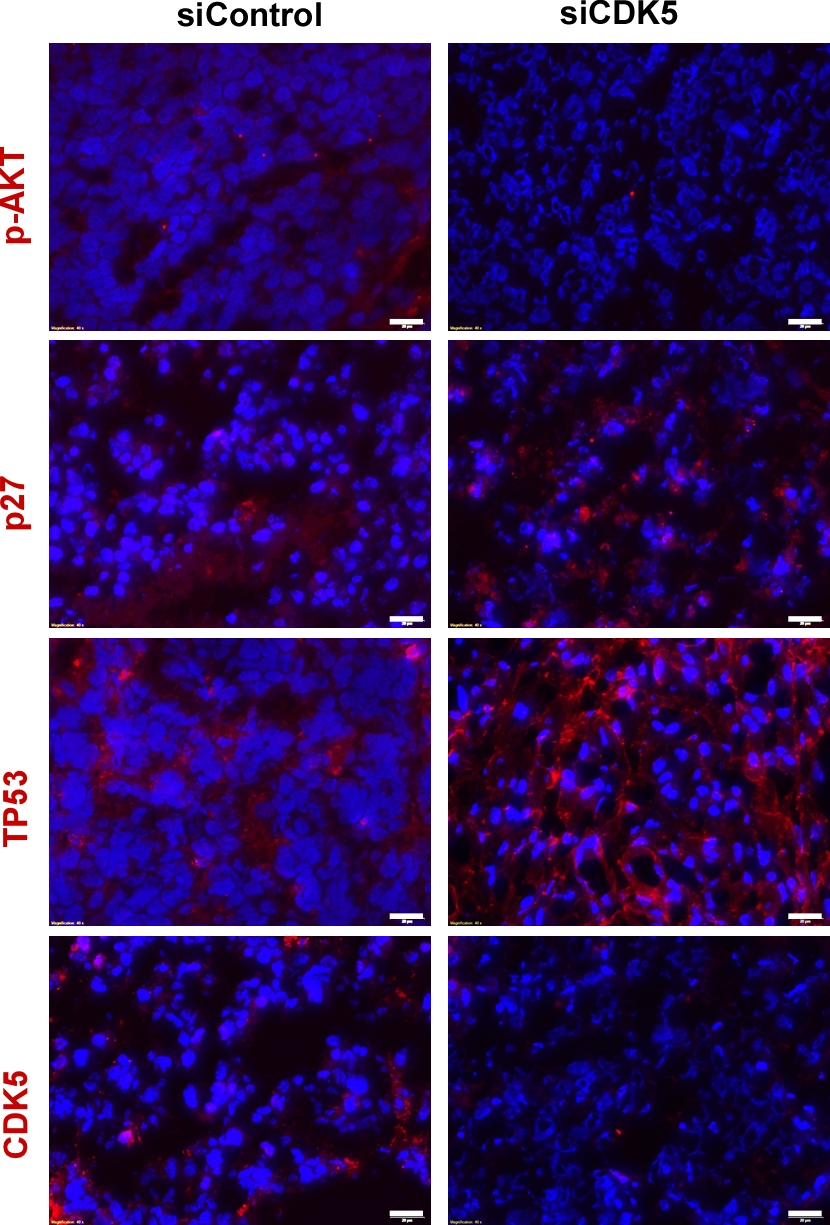


**S5 Fig. CDK5 siRNA increased protein level of p53, p27 and inhibited activated AKT in ovarian cancer xenografts.** Tumor Xenografts (from Figure 6A A2780 experiment) were excised and then embedded in OTC. Immunofluorescence staining of endogenous p-AKT, p27, TP53 and CDK5 were analyzed by confocal microscopy. Scale bars: 20 μm.
