# Supplementary material for: CDK5 Regulates Paclitaxel Sensitivity in Ovarian Cancer Cells by Modulating AKT Activation, p21Cip1- and p27Kip1-Mediated G1 Cell Cycle Arrest and Apoptosis
Source: PLoS One. 2015 Jul 6;10(7):e0131833. doi: 10.1371/journal.pone.0131833 (PMC4492679; doi:10.1371/journal.pone.0131833)
Supplement: S6 Fig — (DOCX) [file pone.0131833.s007.docx]

**

**

**S6 Fig. Increased expression of CDK5 mRNA in ovarian cancers**. (A) CDK mRNA level from high grade serous cancers in the TCGA assayed with Agilent and Affymetrix chips (http://cancergenome.nih.gov). (B) CDK5 expression from Oncomine - Welsh OVCA (https://www.**oncomine**.org).
