# Supplementary material for: CDK5 Regulates Paclitaxel Sensitivity in Ovarian Cancer Cells by Modulating AKT Activation, p21Cip1- and p27Kip1-Mediated G1 Cell Cycle Arrest and Apoptosis
Source: PLoS One. 2015 Jul 6;10(7):e0131833. doi: 10.1371/journal.pone.0131833 (PMC4492679; doi:10.1371/journal.pone.0131833)
Supplement: S7 Fig — (DOCX) [file pone.0131833.s008.docx]

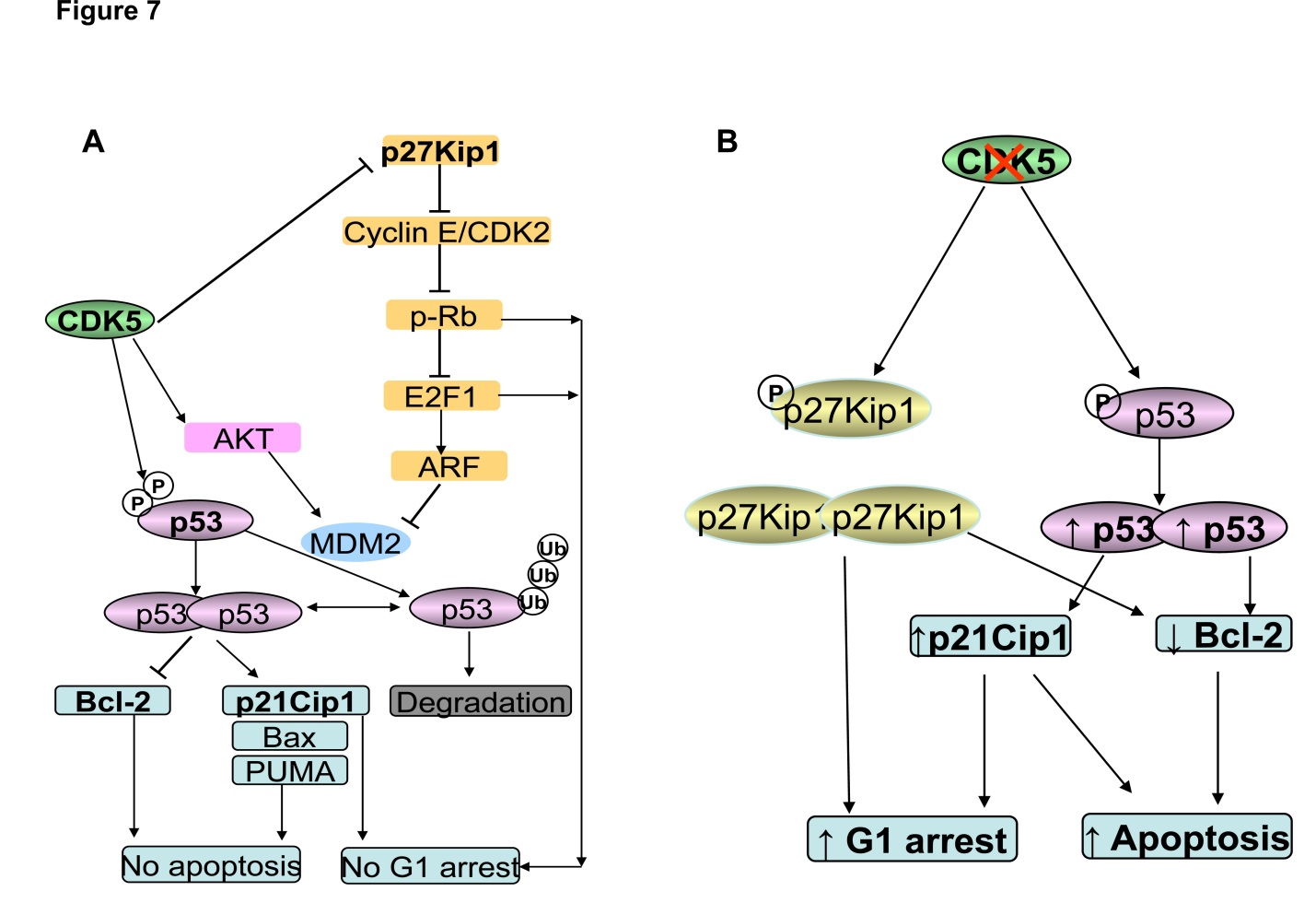


**S7 Fig.** **CDK5 regulates apoptosis and G1 arrest in ovarian cancer cells with wild-type TP53**. CDK5 knockdown induced G1 arrest of the cell cycle and apoptotic cell death, associated with significant induction of p53, p21^Cip1^ and p27^Kip1^ protein.
